# Efficient patient care in the digital age: impact of online appointment scheduling in a medical practice and a university hospital on the “no-show”-rate

**DOI:** 10.3389/fdgth.2025.1567397

**Published:** 2025-05-02

**Authors:** Paola Kammrath Betancor, Daniel Boehringer, Jens Jordan, Charlotte Lüchtenberg, Marcus Lambeck, Manuel Christoph Ketterer, Thomas Reinhard, Michael Reich

**Affiliations:** ^1^ Eye Center, Medical Center—University of Freiburg, Faculty of Medicine, University of Freiburg, Freiburg, Germany; ^2^ Augenärzte am Städel, Frankfurt am Main, Germany; ^3^ Department of Otorhinolaryngology, Medical Center—University of Freiburg, Faculty of Medicine, University of Freiburg, Freiburg, Germany

**Keywords:** efficient patient care, online appointment scheduling, ophthalmology, private practice, university hospital

## Abstract

**Background:**

Online appointment scheduling (OAS) increases patient satisfaction and enables more efficient care.

**Method:**

A retrospective study in an ophthalmology practice and an ophthalmology university hospital. Over 20 months, all booked practice-appointments before and after OAS implementation were recorded. Rates of cancellations/rescheduling and unexcused absences (“no-shows”) were compared. During the same period, OAS usage, no-show rates, and related factors were analyzed in the hospital.

**Results:**

During the observation period, 16,894 appointments were booked in the practice and 81,173 in the hospital. In both, the rate of appointments scheduled via OAS increased continuously, with an average rate of 22.8% in the practice and 7.2% in the hospital. The no-show rate in the practice was lower for appointments booked online compared to those booked offline (median (x¯) 1.8% vs. 5.9%, *p* < 0.0001), whereas it was higher in the hospital (x¯ 14.3% vs. 11.2%, *p* < 0.0001). Regular consultations and SMS reminders were most effective in reducing no-shows in the hospital (Odds Ratio (OR) 0.40 and OR 0.93). The implementation of OAS in the practice reduced the rates of unused appointments (x¯ 22.7% vs. 10.3%, *p* < 0.0001) and never booked appointments (x¯ 8.6% vs. 1.6%, *p* < 0.0001), thereby increasing the utilization of available appointments (*p* < 0.0001).

**Conclusion:**

OAS improves flexibility and resource use in the practice. In the hospital, SMS reminders mostly reduce no-shows, prompting development of a comprehensive reminder model.

## Introduction

The ongoing digitalization is not only transforming our daily life but also the healthcare sector. Online appointment scheduling (OAS) is gaining importance, as it optimizes processes, improves patient satisfaction, and facilitates better resource utilization (1, 2).

Physician practices are typically reachable by phone only during office hours, which ties up personnel resources, among other things (3). University hospitals, on the other hand, are open 24/7 and often experience high patient volumes with occasionally long waiting times. Traditional appointment scheduling presents significant operational challenges: reliance on telephone communication restricts access to office hours, manual coordination consumes considerable personnel resources leading to potential bottlenecks and errors, and inflexibility often results in long patient waiting times and inefficient use of clinical slots (3, 4). Online Appointment Scheduling (OAS) directly addresses these challenges by providing patients with 24/7 booking accessibility, automating the scheduling process to free up staff time, offering enhanced flexibility for patients to manage their appointments (e.g., rescheduling or cancellations), and enabling features like automated reminders. These capabilities aim to improve overall system efficiency, optimize resource utilization, and enhance patient satisfaction (1, 2, 5). A Canadian study demonstrated that patients particularly value the flexibility, time savings, and automatic reminders provided by OAS, which reduce missed appointments (5).

Unannounced missed appointments (no-shows) place a burden on the healthcare system, as they decrease the efficiency of physician practices and hospitals. A Chinese study using the data of the outpatient department at a general hospital analyzed key factors contributing to missed online-scheduled appointments. Of 48,777 scheduled appointments, the no-show rate was 15%. No correlation was found between no-shows and gender, day of the week, or appointment fees, but a positive association was observed with a history of missed appointments (6). A review of 105 studies including multiple types of specialties and different types of clinics identified an average no-show rate of 23%. Higher no-show rates were correlated with longer lead times, previous no-shows, younger patient age, lower socioeconomic status, greater distance from the clinic, and lack of private insurance (7).

In a preceding pilot study, we analyzed all online and offline booked appointments over 12 weeks in an ophthalmology practice. Patients typically rescheduled or canceled appointments 24–48 h in advance using OAS, allowing the practice to reassign freed-up slots, thereby increasing efficiency (8).

While OAS is increasingly adopted, detailed comparative analyses across different healthcare settings (private practice vs. university hospital) within the same specialty, particularly examining under-reported efficiency metrics alongside no-show rates, remain scarce.

This study aimed to analyze how OAS contributes to efficient patient care. Specifically, we first compared the no-show rate in a private practice before and after implementing OAS. Second, we examined OAS usage patterns, no-show rates, and associated influencing factors within a university hospital setting.

## Methods

### Study design

This is a retrospective, two center observational study, conducted in a private ophthalmology practice in collaboration with an ophthalmology university hospital. Following a written request to the Ethics Committee of the responsible State Medical Association of Hessen, Germany, prior to the study initiation, the committee informed us that, in accordance with § 15 Abs. 1 BO, an ethics vote and written informed consent from participating subjects or their legally authorized representatives are not required for this study (ethical approval number: 2024-3837-AF), as § 15 Abs. 1 BO exempts retrospective analyses of pseudonymized or anonymized routine healthcare data under these conditions. Under the updated Health Data Utilization Act (GDNG) § 6, as of 26.03.2024, legally stored data may be processed for medical research purposes.

### Study population

The study cohort included all patients of the practice and the university hospital who scheduled appointments, both online and offline, during the observation period.

### Inclusion and exclusion criteria

The focus of the data collection was to record all appointments booked online and offline during the observation period to provide a complete representative picture of the use of the OAS system. Therefore, all appointments were included in the study without exception.

### Online appointment scheduling practice

The OAS was implemented in the practice on 01.03.2023 and is managed through a commercially available program. For competitive reasons, the name of the company is not disclosed. The OAS calendar is integrated with the practice's patient record software and is also used for appointments scheduled via phone, email, or in person, ensuring that appointment overlaps are avoided. The calendar was tailored to the practice's office hours. Further details can be found in our pilot study (8).

### Online appointment scheduling university hospital

The OAS system in the university hospital is self-developed. Patients can submit their contact information and request an appointment via a contact/appointment form on the ophthalmology department's homepage at the University Hospital Freiburg, Germany. They are then assigned an appropriate appointment. Due to the wide range of specialized consultations, unlike in the practice setting, patients cannot directly book their preferred appointments. Each request is triaged internally by specialists to determine urgency or to refer the patient back to their treating ophthalmologist. Processing can take up to 7 business days. This necessary triage step, inherent to tertiary care centers managing complex referrals and ensuring patients reach the correct sub-specialist, contributes to the processing time observed. Patients can also use the online form to request appointment cancellations or rescheduling, which are also triaged internally and handled asynchronously. Responses are communicated to patients through a web form, with a cryptographic access link sent via SMS or Quick-Response code.

### SMS reminder

In the practice, patients receive an SMS reminder 24 or 48 h prior to the scheduled appointment, depending on the booking timeframe, through the OAS system. At the university hospital, SMS reminders are sent 7 days before the scheduled appointment via the self-developed OAS system.

### Data collection

#### Observation time

The data collection period extended from 01.09.2022–30.04.2024, allowing for a comparison before and after the implementation of OAS in the practice on 01.03.2023. One of the physicians has been working in the practice since 01.07.2022, so data prior to 01.09.2022 was not representative. This period (September 2022 to February 2023) served as the crucial baseline or control period for the practice setting, allowing for a direct before-and-after comparison to assess the specific impact of OAS implementation on efficiency metrics. While OAS was already operational in the university hospital prior to September 2022, we collected data from the same observation period (01.09.2022–30.04.2024) for both the practice and the hospital. This ensures a consistent timeframe for analyzing trends and allows for comparison of system usage patterns and outcomes (like seasonality effects on no-shows) between the two settings during the defined period.

#### Practice

In the practice, data on all scheduled appointments for three adult consultations were collected during the observation period, differentiated between online and offline booked appointments, as well as patient attendance or absence with or without notification. Additionally, the number of appointment slots that were never booked (meaning appointment slots that always stayed empty), as well as the number of booked but finally unused appointments (due to rescheduling, cancellation, no-show) that could not be filled, were recorded. A “no-show” was defined as an unexcused absence of a patient.

#### University hospital

For the the patient's age (in years), and gender were recorded. Additionally, distinctions were made between various consultation categories analyzed for no-show risk: Private consultations, Intravitreal Injection (IVI) clinic, Neuro-ophthalmology and Pediatrics (NP) clinic, and all other “regular” (general or other subspecialty) ophthalmology consultations combined as the reference group.

### Statistical analysis

GraphPad PRISM (GraphPad Software, Version 8, San Diego, US) was used for data analysis. For descriptive analysis median (x¯) and range was calculated. The chi-square test was employed to compare booking behaviors between groups: appointment cancellations/rescheduling for online vs. offline booked appointments, no-shows for online vs. offline booked appointments, and unused/never booked appointments before vs. after OAS implementation. Significance was defined as *p* < 0.05.

For the practice, due to the economic sensitivity of the data, no distinction was made between patients with public or private insurance. To assess the correlation between practice efficiency (the proportion of unused appointments) and the rate of online-scheduled appointments, a Spearman rank correlation was performed, and a non-linear regression analysis was applied.

For the statistical analysis of factors associated with no-shows in the university hospital, a multifactorial logistic regression analysis was conducted to calculate the odds ratio (OR), including the 95% confidence interval (95% CI), for all covariates considered together. These calculations were performed using the R system, version 4.1.3 (9).

## Results

### Demand of online appointment scheduling

**Figure 1:**
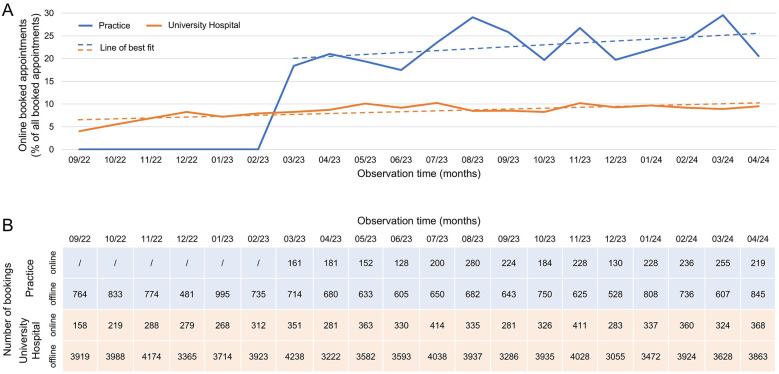
Utilization of online appointment scheduling in practice and university hospital. **(A)** Percentage and **(B)** absolute number of appointments booked via online appointment scheduling relative to all scheduled appointments during the observation period. Line of best fit for practice (blue): *Y* = 0,37 *X* + 17,7, *R*^2^ = 0,15; Line of best fit for university hospital (orange):: *Y* = 0,12 *X* + 13,1, *R*^2^ = 0,24.

Interest in online appointment scheduling (OAS) has been steadily increasing both in the practice and the university hospital (Figure 1). Of the total 12,312 appointments scheduled at the practice since the introduction of OAS, 22.8% (2,806 appointments) were booked online. A total of 4,582 appointments took place before the introduction of OAS from September 2022–February 2023.

At the university hospital, where the majority of appointments are directly requested by specialists, 7.2% (6,288 of 81,173 appointments) were booked online.

### Impact of online appointment scheduling and other factors on the no-show rate

We analyzed the impact of booking method (online vs. offline) and, particularly in the hospital setting, other factors including seasonality (month), patient gender, patient age, use of SMS reminders, and type of specialty consultation on no-show rates.

**Figure 2:**
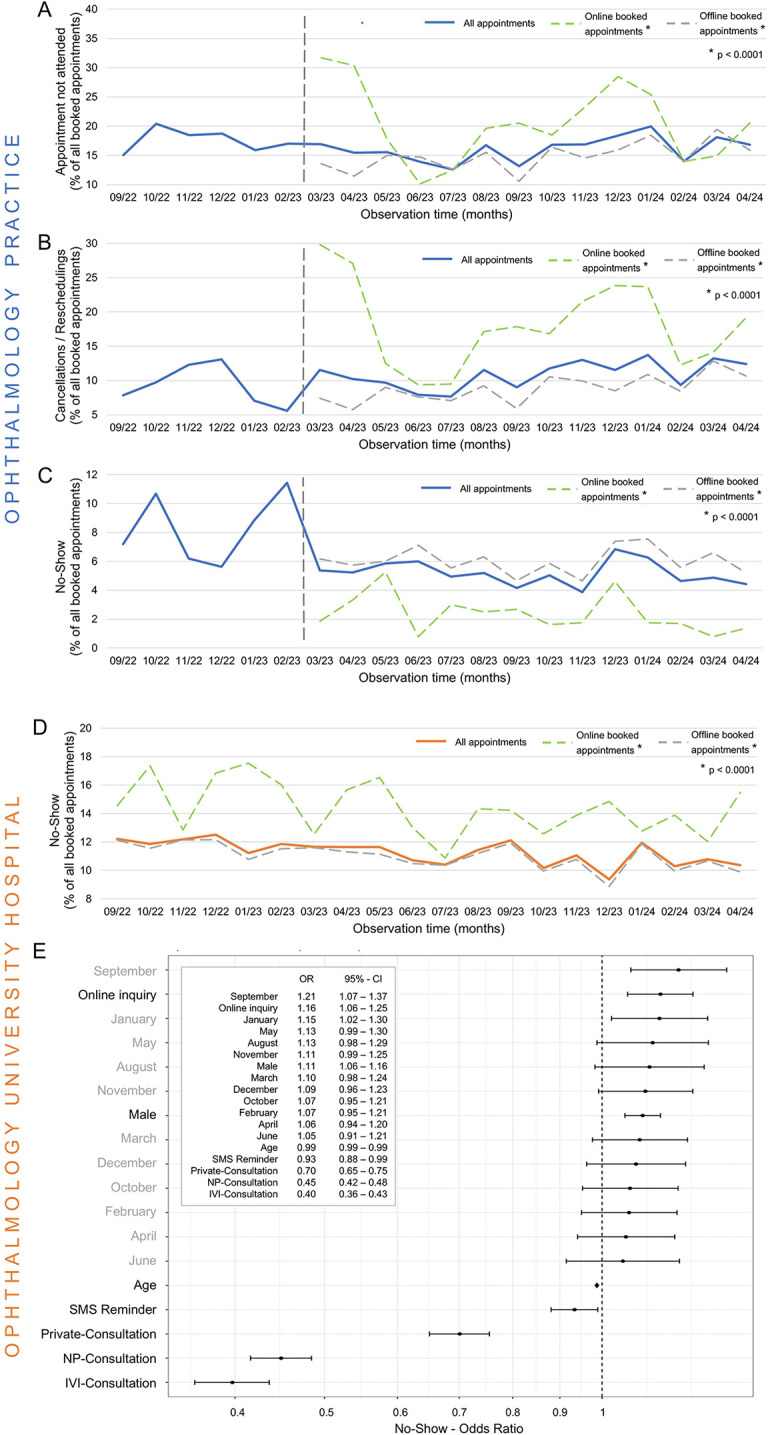
No-Show rates for online vs. Offline Booked Appointments. **(A)** Percentage of missed appointments, **(B)** percentage of canceled/rescheduled appointments, and **(C)** percentage of no-shows relative to all (blue), exclusively online (green), and exclusively offline (light gray) booked appointments in the ophthalmology practice. The vertical dark gray line marks the implementation of online appointment scheduling in the practice. Statistical comparisons between online and offline booked appointments were made using the chi-square test. **(D)** Percentage of no-shows relative to all (orange), exclusively online (green), and exclusively offline (light gray) booked appointments at the University Hospital, Department of Ophthalmology. Statistical comparisons between online and offline booked appointments were made using the chi-square test. **(E)** Statistical representation of the odds ratios (OR) for various factors associated with a no-show appointment at the University Hospital, Department of Ophthalmology. Eleven months (light gray) are compared to July (this month is excluded from the list, as it exhibited the lowest no-show potential). Online requests are compared to appointments scheduled by a specialist. Male patients are compared to female patients. Age is presented relative to years of life. Appointments with SMS reminders are compared to those without reminders. Specialized consultations (Private, NP, neuroophthalmology and pediatrics; IVI, intravitreal injections) are compared to all other regular patients.

In the practice, the overall rate of appointments not attended as originally scheduled (i.e., including cancellations, rescheduling, and no-shows) was higher for online bookings compared to offline bookings (up to 31.7% vs. 19.4%, *p* < 0.0001, Figure 2A). The ability to reschedule or cancel appointments was utilized more often for online-booked appointments compared to offline bookings (up to 29.8% in March 2023 vs. 12.9% in March 2024, *p* < 0.0001, Figure 2B). However, the no-show rate for online-booked appointments was significantly lower than for offline booked appointments (x¯ 1.8%, range 0.8%–5.3%, vs. x¯ 5.9%, range 4.6%–7.6%, *p* < 0.0001, Figure 2C).

In contrast, at the university hospital, the no-show rate for online-scheduled appointments was higher (x¯ 14.3%, range 10.9%–17.5%, vs. x¯ 11.2%, range 8.9%–12.2%, *p* < 0.0001, Figure 2D, OR 1.16, Figure 2E) and exhibited clear fluctuations. From September 2022–February 2024, a slight decline in the no-show rate was observed (12.2% vs. 10.4%, Figure 2D). Seasonally, July had the lowest no-show potential (Figure 2E), though July was only captured once during the observation period. Male patients had a higher risk of no-shows (OR 1.11, Figure 2E), while the risk decreased with increasing age (OR 0.99, Figure 2E). SMS reminders also reduced the no-show risk (OR 0.93, Figure 2E). The consultation of intravitreal injection (IVI) had the lowest no-show rate of all specialty consultations, followed by the neuro-ophthalmology and pediatric consultation (NP), and the consultation for patients with private health insurance (OR 0.40, 0.45, and 0.70, respectively, Figure 2E).

### Impact of SMS reminders on the no-show rate at the university hospital

**Figure 3:**
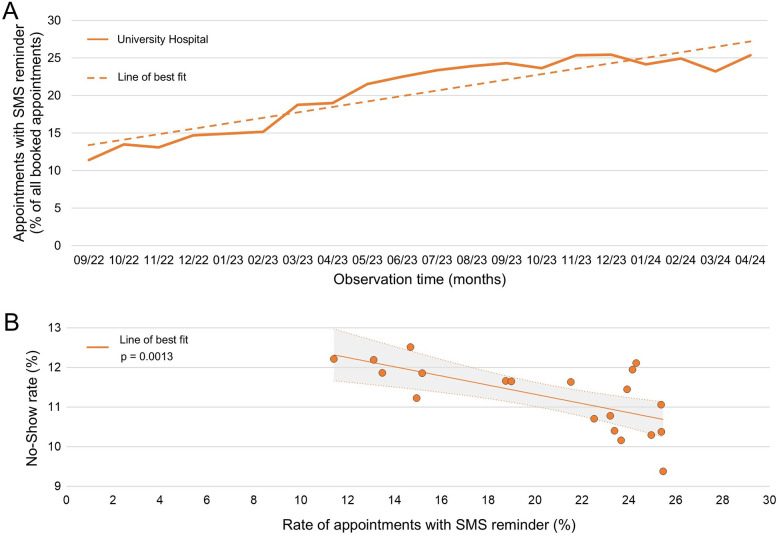
Impact of SMS reminders on No-show rates at the university hospital. **(A)** Percentage of appointments with SMS reminders relative to all scheduled appointments during the observation period. Line of best fit: *Y* = 0.76 *X* + 12.5, *R*² = 0.85. **(B)** Correlation between the percentage of appointments with SMS reminders and the no-show rate percentage. Spearman *r* = −0.67; 95% confidence interval: −0.86 to −0.31, *p* = 0.0013. The trend curve was created using linear regression analysis, *R*² = 0.44.

The use of SMS reminders at the university hospital increased from 11.4% in September 2022–25.4% in April 2024 (Figure 3A). This rise in SMS reminders correlated with a decrease in the no-show rate (*p* = 0.0013, Figure 3B).

### Impact of online appointment scheduling on the practice’s efficiency

**Figure 4:**
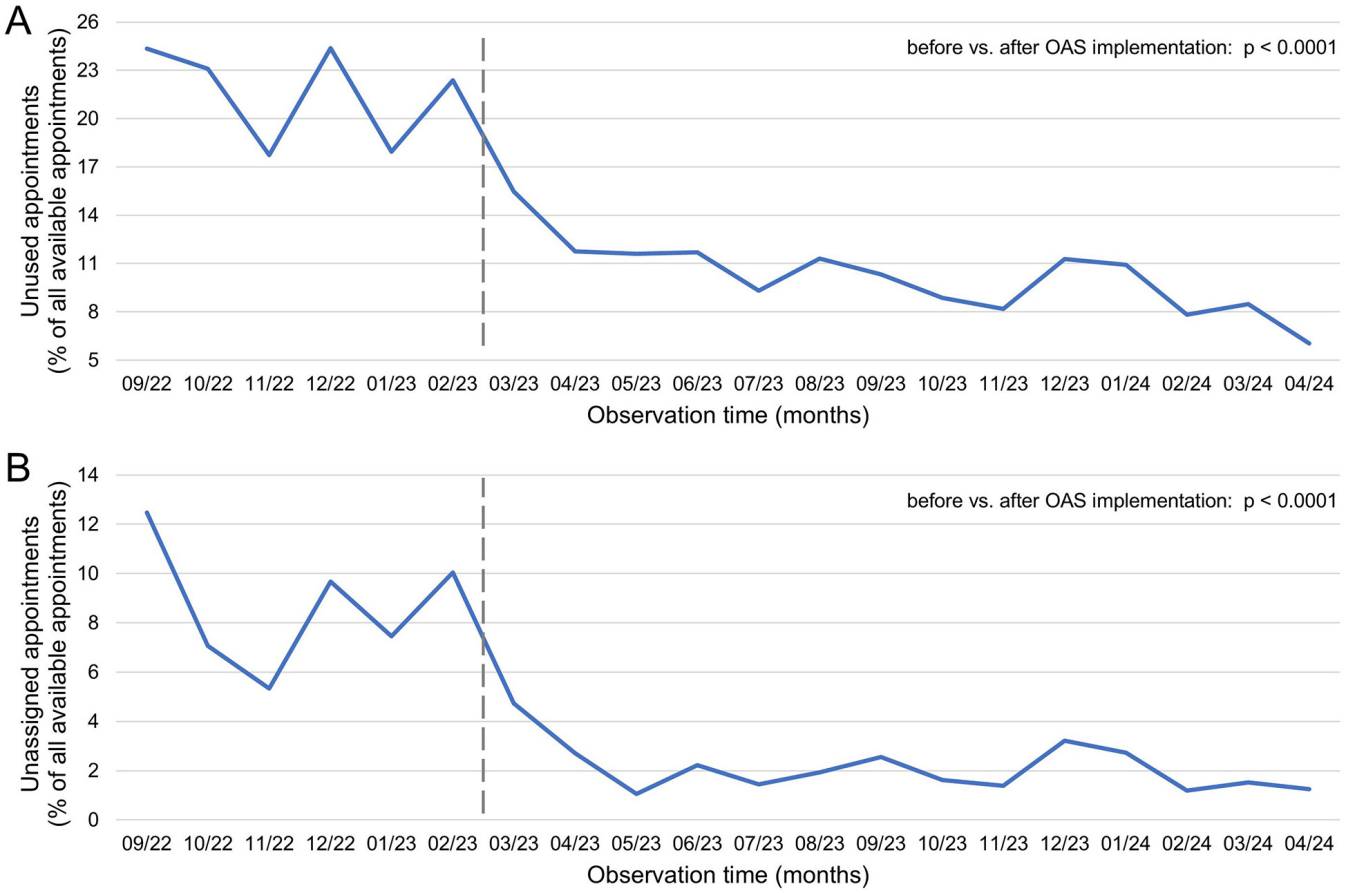
Efficiency improvement through enhanced resource utilization following the Introduction of online appointment scheduling in a medical practice. **(A)** Percentage of unused appointments (due to cancellations/reschedulings/no-shows/never booked appointments) and **(B)** specifically, the percentage of never booked appointments relative to all available appointments. The vertical dark gray line marks the introduction of online appointment scheduling in the practice. Statistical comparisons between the pre- and post-introduction phases were made using the chi-square test.

Before the introduction of OAS at the practice, the rate of unused appointments showed clear fluctuations, with a range of 6.7% (from 17.7%–24.4%, Figure 4A), and never-scheduled appointments varied between 5.3% and 12.5% (Figure 4B). After the implementation of OAS, these rates decreased significantly, with a median rate of unused appointments before vs. after implementation of 22.7% vs. 10.3%, (range 17.7%–24.4% vs. 7.8%–11.8%, *p* < 0.0001, Figure 4A) and a median rate of never-scheduled appointments of 8.6% vs. 1.6% (range 5.3%–12.5% vs. 1.1%–3.2%, *p* < 0.0001, Figure 4B). After implementation of OAS the rate of unused appointments continuously further declined (from 11.8% in April 2023–6.0% in April 2024), and the range of never-scheduled appointments narrowed to 2.1% (range from 1.1%–3.2%). March 2023 was excluded from this analysis, as the introductory phase of OAS was considered non-representative.

**Figure 5:**
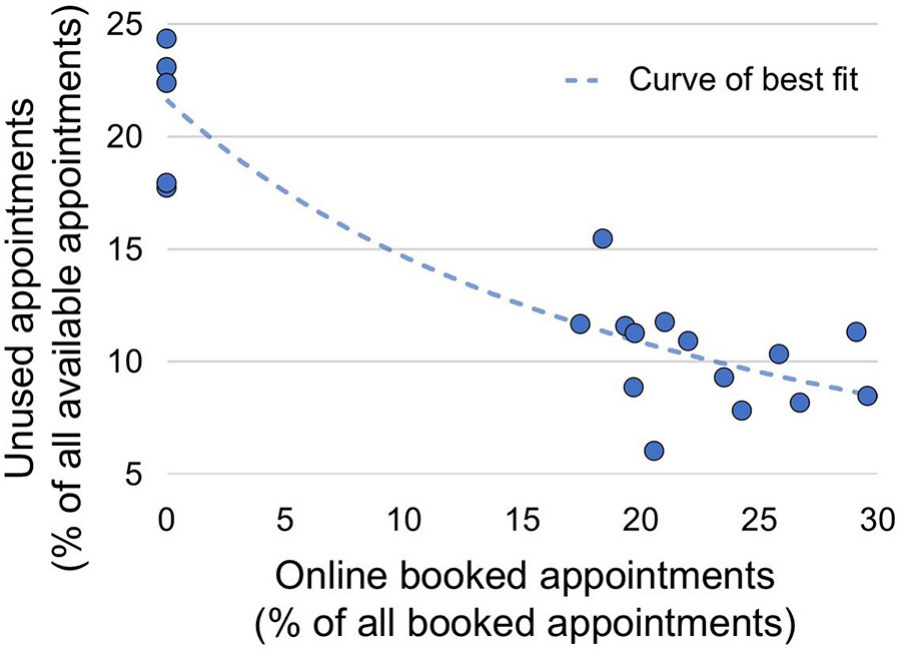
Correlation between increased efficiency and a higher utilization of available appointments with a growing share of online booked appointments. Correlation between the percentage of appointments booked via online appointment scheduling (Figure 1, Practice) and the percentage of unused appointments (Figure 4A). Spearman *r* = −0.82; 95% confidence interval: −0.93 to −0.58, *p* < 0.0001. The curve of best fit was created using nonlinear regression analysis, *R*² = 0.87. A hypothetical intersection at *X* = 100, *Y* = 2.36 was assumed. The *Y*-value corresponds to the average no-show rate for online-booked appointments between March 2023 and April 2024 (Figure 2C).

As the utilization of OAS increased, appointment occupancy rates rose, leading to improved efficiency in the practice (*p* < 0.0001, Figure 5).

## Discussion

This study provides a unique comparison of OAS implementation in two distinct ophthalmology settings—a private practice and a university hospital—analyzing not only no-show rates but also broader efficiency metrics like unused and never-booked appointments over a substantial 20-month period.

In recent years, online appointment scheduling (OAS) has become an integral part of patient care (2). This study demonstrates that OAS contributes to the efficient use of resources in the healthcare sector.

### Increasing demand for online appointment scheduling

OAS is becoming particularly relevant in medical practices (10, 11), and is also increasingly being used by patients in hospitals (12), as illustrated in Figure 1. A survey conducted by the Digital Association Bitkom in November 2022 revealed that 33% of Germans book their medical appointments online, and 34% are considering doing so (13). Furthermore, 66% believe that all healthcare facilities should offer OAS, and 22% specifically choose practices that provide this service.

### Impact of online appointment scheduling and other factors on no-show rates

Commercially available OAS programs allow patients to easily reschedule or cancel their appointments (5, 14). In practice, appointments booked online are more frequently missed (Figure 2A) or explicitly rescheduled or canceled (Figure 2B). As described in our pilot study, cancellations or reschedulings typically occur 24–48 h before the appointment (8). This flexibility is correlated with a lower no-show rate for online bookings (15), enabling timely reallocation of available slots, which is financially beneficial for medical practices (2, 16).

Most appointments at university hospitals are requested directly by specialists for their patients. Since patients in these hospital settings cannot easily cancel or reschedule appointments autonomously via a direct booking interface, unlike the OAS model used in the practice, the no-show rate for online-booked appointments is higher (Figures 2C,D). This difference underscores how the specific implementation of OAS (direct booking vs. request/triage system) and the level of patient autonomy significantly influence outcomes like no-show rates. Interestingly, the lowest no-show potential, in terms of seasonality, was observed in July (Figure 2E), rather than in the winter months, as one might expect (17). Regarding specific consultations, the consultation of intravitreal injection (IVI) at the university hospital showed the lowest no-show rates, likely due to the regularity of the consultations, typically scheduled every four weeks. Various studies suggest that a shorter interval between appointment request and appointment date leads to a lower no-show rate (6, 18). The observed lower risk of no-shows with increasing patient age aligns with some literature suggesting younger patients may have higher no-show rates, potentially reflecting different life priorities or stability. Appointments in the neuroophthalmology and pediatric consultation (NP) are rarely missed, as both parents are often present, allowing for more precise scheduling. The slightly higher risk for male patients is also seen in some studies, though reasons are complex and may relate to differing healthcare engagement patterns. Privately insured patients tend to miss appointments less frequently, which may be attributed to a higher adherence to health-related commitments (19, 20).

### Positive impact of SMS reminders on the no-show rate at the university hospital

SMS reminders are a simple tool that notify patients of their upcoming appointments and have thus been shown to reduce the no-show rate in our analysis (Figure 3). Kheirkhah et al. studied the prevalence, predictors, and economic consequences of patient no-shows in 10 American clinics, each with a different specialty. In their study, centralized telephone reminders reduced the no-show rate only slightly, from 16.3%–15.8% (21). However, a systematic review of 26 studies including multiple specialties in different countries by Robotham et al. revealed that patients reminded of their appointments were 23% more likely to attend (22).

### Positive impact of online appointment scheduling on practice efficiency

The introduction of OAS, primarily through the significant reduction in unused or unallocated appointments, has markedly increased the efficiency of the medical practice presented in this study (Figure 4). Figure 5 highlights the positive correlation between efficiency improvement and the rate of online-booked appointments. The reduced variability in the rate of missed or unused appointments following OAS implementation (Figure 4) enables more precise personnel planning, thereby lowering staffing costs. Additionally, the automation of the appointment scheduling process reduces administrative burden and costs, which are otherwise limited by telephone line capacity and appointment schedulers (23). Comparable results have already been observed in industries outside healthcare. For instance, in the transportation and hospitality sectors, the use of consumer-based online reservation systems has been shown to improve operations (24), profitability (25), customer loyalty (26) and wait times (27). Concerns expressed by some physicians in the literature regarding a potential imbalance between the costs and benefits of OAS (10, 28) are unfounded based on the findings of this study.

### Strengths of the study

This study has several strengths, including its two-center design allowing direct comparison between a private practice and a university hospital within the same specialty. The large dataset collected over 20 months, encompassing periods before and after OAS implementation in the practice, enables robust analysis of its impact. Furthermore, the examination of specific efficiency metrics like “unused” and “never booked” appointments provides a more comprehensive assessment of resource utilization than focusing solely on no-show rates.

### Limitations

This study is based on data from an ophthalmology practice and an ophthalmology university hospital. While the core principles observed (e.g., patient demand for digital access, impact of reminders) likely apply broadly, the magnitude of the effects of OAS on no-show rates and efficiency might differ in other specialties depending on patient populations, appointment types, and specific OAS workflows. Patient cohorts from other medical specialties may demonstrate different behaviors with OAS due to varying demographic compositions, which may limit the generalizability of these findings to other medical fields.

Regarding the practice data, no distinction was made between patients with statutory or private insurance, as this information involves sensitive business data. Additionally, no demographic data were collected. Thus, it is not possible to evaluate whether patient-related factors such as gender or age have an impact on the no-show rate.

Reliance on OAS introduces vulnerabilities related to technology, including potential system outages, software integration challenges, data privacy, and cybersecurity risks, which were not evaluated in this study.

Due to the specialized nature of the consultations within a single medical specialty, it is not possible to offer OAS in a university hospital as in a medical practice. For the same reason, patients cannot directly reschedule or cancel their appointments. Consequently, the self-developed OAS system in the university hospital is used less frequently than in the medical practice. This shows that simpler direct-booking OAS models are less suitable for scenarios requiring complex triage, multidisciplinary coordination, or specific sequences of appointments. Furthermore, due to this distinction, our data from the private ophthalmology practice and the university hospital cannot be fully compared on a one-to-one basis.

### Outlook

The finding that SMS reminders reduce the no-show rate in the university hospital has prompted the development of a system in which all patients — new and returning, regardless of whether they book appointments online or offline — will be asked at registration if they wish to receive SMS reminders. In the medical practice, patients who do not book their appointments online are already being offered the option of receiving SMS reminders.

Building on the observed effectiveness of SMS reminders in the hospital setting, concrete next steps could include conducting interviews and surveys with diverse patient groups and healthcare staff to gain deeper insights into the barriers (e.g., digital literacy, usability issues) and facilitators influencing OAS adoption, satisfaction, and impact on patient experience. Furthermore, rigorous economic evaluations should be performed to compare the implementation and maintenance costs of OAS systems and integrated reminder strategies against the potential savings derived from reduced no-show rates and improved operational efficiency.

## Conclusion

The introduction of OAS in medical practices provides patients with greater flexibility in their appointment scheduling and has led to a reduction in no-shows, better utilization of available appointments, and thus more efficient resource use as the proportion of online-booked appointments has increased. In contrast, the use of online appointment scheduling in university hospitals is less relevant due to the referral-based system and the lack of direct booking options for patients. However, SMS reminders still reduce the no-show rate in these settings. These findings underscore the context-dependent benefits of OAS and highlight the consistent value of reminders, so that an appointment reminder model is currently being developed for all appointments at the Department of Ophthalmology at the University Hospital Freiburg. Further research, as outlined in the Outlook, is needed to optimize these digital tools, particularly through comparative studies, cost-effectiveness analyses, and the development of more sophisticated, potentially personalized, reminder strategies to maximize efficiency and patient access across diverse healthcare settings.

While healthcare systems and specific OAS implementations vary globally, the core principles demonstrated here—patient demand for digital convenience, the potential for OAS to enhance efficiency (particularly in primary/private care), and the utility of automated reminders—possess broad international relevance, informing efforts to optimize patient access and resource allocation worldwide.

## Data Availability

The original contributions presented in the study are included in the article/Supplementary Material, further inquiries can be directed to the corresponding author.

## References

[1] Prinz S, Rashid A. Online-Terminmanagement: Viele Potenziale für Arztpraxen. Ärzteblatt DÄG Redaktion Deutsches. Deutsches Ärzteblatt (2015). Available at: https://www.aerzteblatt.de/archiv/169213/Online-Terminmanagement-Viele-Potenziale-fuer-Arztpraxen (Accessed April 26, 2025).

[2] Woodcock EW. Barriers to and facilitators of automated patient self-scheduling for health care organizations: scoping review. J Med Internet Res. (2022) 24(1):e28323. 10.2196/28323 35014968 PMC8790681

[3] Czeschik C. Praxisorganisation: Online-Terminvereinbarung in der Arztpraxis. Ärzteblatt DÄG Redaktion Deutsches. Deutsches Ärzteblatt (2021). Available at: https://www.aerzteblatt.de/archiv/218967/Praxisorganisation-Online-Terminvereinbarung-in-der-Arztpraxis (Accessed April 26, 2025).

[4] Siegel H Böhringer D Wacker K Niedenhoff PJL Mittelviefhaus H Reinhard T. Duration of consultations in an outpatient ophthalmology unit. Dtsch Arztebl Int. (2023) 120(27–28):481–2. 10.3238/arztebl.m2023.0037 37661318 PMC10487673

[5] Paré G Trudel MC Forget P. Adoption, use, and impact of e-booking in private medical practices: mixed-methods evaluation of a two-year showcase project in Canada. JMIR Med Inform. (2014) 2(2):e24. 10.2196/medinform.3669 25600414 PMC4288107

[6] Su W Zhu C Zhang X Xie J Gong Q. Who misses appointments made online? Retrospective analysis of the outpatient department of a general hospital in Jinan, Shandong province, China. Risk Manag Healthc Policy. (2020) 13:2773–81. 10.2147/RMHP.S280656 33273875 PMC7708679

[7] Dantas LF Fleck JL Cyrino Oliveira FL Hamacher S. No-shows in appointment scheduling—a systematic literature review. Health Policy. (2018) 122(4):412–21. 10.1016/j.healthpol.2018.02.002 29482948

[8] Betancor PK Jordan J Boehringer D Lüchtenberg C Lambeck M Ketterer MC Efficient patient care in the digital age: online appointment scheduling in an ophthalmology practice. Digital Health. (2024) 10:20552076241287083. 10.1177/20552076241287083 39430692 PMC11489984

[9] R. The R Project for Statistical Computing. Available at: https://www.r-project.org/ (Accessed April 26, 2025).

[10] Obermann K, Müller P. Ärzte im Zukunftsmarkt Gesundheit. (2010). Available at: https://epub.sub.uni-hamburg.de/epub/volltexte/2011/11759/pdf/Studie_Aerzte_im_Zukunftsmarkt_Gesundheit_2010.pdf (Accessed April 26, 2025).

[11] Mazaheri Habibi MR Abadi FM Tabesh H Vakili-Arki H Abu-Hanna A Eslami S. Evaluation of patient satisfaction of the status of appointment scheduling systems in outpatient clinics: identifying patients’ needs. J Adv Pharm Technol Res. (2018) 9(2):51–5. 10.4103/japtr.JAPTR_134_18 30131937 PMC6078003

[12] Volk AS Davis MJ Abu-Ghname A Warfield RG Ibrahim R Karon G Ambulatory access: improving scheduling increases patient satisfaction and revenue. Plast Reconstr Surg. (2020) 146(4):913–9. 10.1097/PRS.0000000000007195 32970013

[13] e.V B. Ein Drittel vereinbart Arzttermine per Internet | Presseinformation | Bitkom e. V (2022). Available at: https://www.bitkom.org/Presse/Presseinformation/Ein-Drittel-Arzttermine-per-Internet (Accessed April 26, 2025).

[14] Chiereghin A Pizzi L Squillace L Bazzani C Roti L Mezzetti F. The positive effect of an online appointment portal on a breast cancer screening program. Appl Clin Inform. (2023) 14(4):609–19. 10.1055/s-0043-1769910 37557889 PMC10412065

[15] Habibi MRM Mohammadabadi F Tabesh H Vakili-Arki H Abu-Hanna A Eslami S. Effect of an online appointment scheduling system on evaluation metrics of outpatient scheduling system: a before-after MulticenterStudy. J Med Syst. (2019) 43(8):281. 10.1007/s10916-019-1383-5 31300894

[16] Berg BP Murr M Chermak D Woodall J Pignone M Sandler RS Estimating the cost of no-shows and evaluating the effects of mitigation strategies. Med Decis Making. (2013) 33(8):976–85. 10.1177/0272989X13478194 23515215 PMC4153419

[17] Curry EJ Tybor DJ Jonas N Pevear ME Mason A Cipriani LJ An evaluation of risk factors for patient “no shows” at an urban joint arthroplasty clinic. J Am Acad Orthop Surg. (2020) 28(22):e1006–13. 10.5435/JAAOS-D-19-00550 33156587

[18] Giunta D Briatore A Baum A Luna D Waisman G de Quiros FGB. Factors associated with nonattendance at clinical medicine scheduled outpatient appointments in a university general hospital. Patient Prefer Adherence. (2013) 7:1163–70. 10.2147/PPA.S51841 24235820 PMC3826940

[19] Stauder J Kossow T. Selection or better service—why are those with private health insurance healthier than those covered by the public insurance system? Gesundheitswesen. (2017) 79(3):181–7. 10.1055/s-0042-104583 27171730

[20] Kriwy P Mielck A. Persons insured with the German statutory sickness funds or privately insured: differences in health and health behaviour. Gesundheitswesen. (2006) 68(5):281–8. 10.1055/s-2006-926779 16773548

[21] Kheirkhah P Feng Q Travis LM Tavakoli-Tabasi S Sharafkhaneh A. Prevalence, predictors and economic consequences of no-shows. BMC Health Serv Res. (2016) 16(1):13. 10.1186/s12913-015-1243-z 26769153 PMC4714455

[22] Robotham D Satkunanathan S Reynolds J Stahl D Wykes T. Using digital notifications to improve attendance in clinic: systematic review and meta-analysis. BMJ Open. (2016) 6(10):e012116. 10.1136/bmjopen-2016-012116 27798006 PMC5093388

[23] Tang J Yan C Cao P. Appointment scheduling algorithm considering routine and urgent patients. Expert Syst Appl. (2014) 41:4529–41. 10.1016/j.eswa.2014.01.014

[24] Mak HY Rong Y Zhang J. Sequencing appointments for service systems using inventory approximations. M&SOM. (2014) 16(2):251–62. 10.1287/msom.2013.0470

[25] Shugan SM Xie J. Advance pricing of services and other implications of separating purchase and consumption. J Serv Res. (2000) 2(3):227–39. 10.1177/109467050023001

[26] Chiu CK. Understanding relationship quality and online purchase intention in e-tourism: a qualitative application. Qual Quant. (2009) 43(4):669–75. 10.1007/s11135-007-9147-6

[27] Robinson LW Chen RR. Estimating the implied value of the customer’s waiting time. M&SOM. (2011) 13(1):53–7. 10.1287/msom.1100.0304

[28] Zhao P Yoo I Lavoie J Lavoie BJ Simoes E. Web-based medical appointment systems: a systematic review. J Med Internet Res. (2017) 19(4):e134. 10.2196/jmir.6747 28446422 PMC5425771

